# Multi-Targeting Bioactive Compounds Extracted from Essential Oils as Kinase Inhibitors

**DOI:** 10.3390/molecules25092174

**Published:** 2020-05-06

**Authors:** Annalisa Maruca, Delia Lanzillotta, Roberta Rocca, Antonio Lupia, Giosuè Costa, Raffaella Catalano, Federica Moraca, Eugenio Gaudio, Francesco Ortuso, Anna Artese, Francesco Trapasso, Stefano Alcaro

**Affiliations:** 1 Dipartimento di Scienze della Salute, Università “Magna Græcia” di Catanzaro, Campus Universitario “S. Venuta”, Viale Europa, Loc. Germaneto, 88100 Catanzaro, Italy; maruca@unicz.it (A.M.); gcosta@unicz.it (G.C.); catalano@unicz.it (R.C.); ortuso@unicz.it (F.O.); artese@unicz.it (A.A.); 2 Net4Science srl, Università “Magna Græcia” di Catanzaro, Campus Universitario “S. Venuta”, Viale Europa, Loc. Germaneto, 88100 Catanzaro, Italy; rocca@unicz.it (R.R.); lupia@unicz.it (A.L.); federica.moraca@unina.it (F.M.); 3 Department of Experimental and Clinical Medicine, Università “Magna Græcia” di Catanzaro, Campus Universitario “S. Venuta”, Viale Europa, Loc. Germaneto, 88100 Catanzaro, Italy; delialanzillotta@unicz.it (D.L.); trapasso@unicz.it (F.T.); 4 Department of Pharmacy, University “Federico II” of Naples, Via D. Montesano 49, 80131 Naples, Italy; 5 Lymphoma and Genomics Research Program, the Institute of Oncology Research, 6500 Bellinzona, Switzerland; eugenio.gaudio@ior.usi.ch

**Keywords:** essential oils, multi-target, polypharmacology, kinases, anti-cancer, SBVS

## Abstract

Essential oils (EOs) are popular in aromatherapy, a branch of alternative medicine that claims their curative effects. Moreover, several studies reported EOs as potential anti-cancer agents by inducing apoptosis in different cancer cell models. In this study, we have considered EOs as a potential resource of new kinase inhibitors with a polypharmacological profile. On the other hand, computational methods offer the possibility to predict the theoretical activity profile of ligands, discovering dangerous off-targets and/or synergistic effects due to the potential multi-target action. With this aim, we performed a Structure-Based Virtual Screening (SBVS) against X-ray models of several protein kinases selected from the Protein Data Bank (PDB) by using a chemoinformatics database of EOs. By evaluating theoretical binding affinity, 13 molecules were detected among EOs as new potential kinase inhibitors with a multi-target profile. The two compounds with higher percentages in the EOs were studied more in depth by means Induced Fit Docking (IFD) protocol, in order to better predict their binding modes taking into account also structural changes in the receptor. Finally, given its good binding affinity towards five different kinases, cinnamyl cinnamate was biologically tested on different cell lines with the aim to verify the antiproliferative activity. Thus, this work represents a starting point for the optimization of the most promising EOs structure as kinase inhibitors with multi-target features.

## 1. Introduction

Essential oils (EOs), also called volatile or ethereal oils, are aromatic, highly volatile, hydrophobic liquids produced by aromatic plants as secondary metabolites. Nowadays, more than 3000 EOs are known and about 300 are relevant for pharmaceutical, agronomic, food, cosmetic, and perfume industries. EOs can be extracted from several plant organs (i.e. buds, flowers, leaves, stem, twigs, seeds, fruits, roots, wood, or bark) and they are generally obtained by distillation [1]. Each essential oil is a very complex mixture of molecules, containing from 20 to 70 components with a low molecular weight at different concentrations. Many molecules are measurable in traces, while only two or three of them are present with a high percentage (20–70%), thus determining the biological activities of the essential oil. Considering their chemical structures, the EOs constituents are classified as: (1) Terpene hydrocarbons (derived from C5 isoprene unit); (2) terpenoids (terpenes containing oxygen), such as alcohols, ketones, aldehydes, esters, lactones and coumarins; (3) phenylpropanoids and aromatic compounds, derived from phenylpropane. Several factors affect the composition of EOS, such as the plant variety, the growth area, climatic changes, storage conditions and extraction methods. Typically, monoterpenes are the most abundant constituents and, often, the biological activities of EOs have been related to their presence in the phytocomplex [2,3]. Traditionally, EOs have been used for their biological activities including antibacterial, antifungal, sedative, antioxidant, spasmolytic, carminative, hepatoprotective, and analgesic effects. Finally, several studies reported EOs bioactive compounds as potential anti-cancer agents against liver, lung, colon and prostate cancer [4,5]. Different mechanisms of action were proposed to explain the carcinogenic prevention by EOs treatments, such as induction of cell apoptosis, cell cycle arrest, increase of reactive oxygen species and nitrogen levels (ROS/RNS) in cancer cells. In this context, most Protein Kinase targets are being investigated for the cancer treatment, because involved in many processes leading to tumor cell proliferation and survival [6]. Many kinases, with the exception of the atypical kinases, share a conserved catalytic domain, which transfers a phosphate from ATP to a protein target, by modulating several important steps in cellular processes such as growth, differentiation, proliferation, and apoptosis [7]. The main classes of kinase are tyrosine kinases (TKs) and serine–threonine kinases (STKs), which phosphorylate tyrosine and serine or threonine residues on a substrate, respectively. TKs and STKs can be both membrane-bound and nuclear; in addition, TKs can be transmembrane receptors while STKs can be cytoplasmic [8]. In the Oncology Drug Discovery, the current popularity of kinases as drug targets is driven by the convergence of several factors. In particular, the inhibition of kinases activity in normal cells can often be tolerated, presenting a useful therapeutic window for the selective killing of tumor cells [9]. Recently, the anticancer potential of EOs linked to the targeting of kinases has been explored and several studies are now available in the literature [10,11,12,13,14,15,16].

As for computational methods, the identification of new *hit* compounds by a virtual screening (VS) approach can represent a crucial step in early-stage of drug discovery. In fact, theoretical studies, based on chemoinformatics and bioinformatics methods, are capable to speed up the identification of bioactive compounds, by testing with in vitro and in vivo assays only the most promising candidates selected through in silico simulations [17,18,19,20,21,22,23]. In this regard, SBVS is a computational approach useful to identify novel bioactive ligands against a certain target or a set of interesting targets, getting information from the three-dimensional (3D) structures of proteins or nucleic acids, obtained from X-ray or NMR methodologies. For instance, in a previous work, a structure-based approach helped to clarify the binding mode of the natural polyphenol Resveratrol against the serine/threonine-protein kinase Sgk1 in its wild-type and phosphorylated forms [24]. Besides, in recent years, the “one target, one drug” paradigm, that has traditionally ruled drug discovery thinking, has been challenged by the evidence that molecules interact concurrently with multiple targets (polypharmacology phenomenon) [25]. Therefore, computational techniques may predict in silico the theoretical ligand activities profile *versus* a set of targets, thereby potential selectivity issues or multi-target activities may be early identified, rationalizing favorable synergic effects or dangerous side ones, caused by drug binding to unwanted off-targets [26,27,28]. With this purpose, we performed a SBVS by using a database of EOs bioactive components and a dataset of kinase receptors. Docking simulations were carried out and theoretical binding affinities of the ligand-target complexes were taking into account in order to select the most promising compounds and to design potential multi-target agents (MTAs) [17]. In particular, 13 molecules displayed a virtual multi-targeting profile. Among them, two most abundant compounds found in nature, i.e. cinnamyl cinnamate and α-terpinen-7-al were submitted to an Induced Fit Docking (IFD) protocol, in order to better characterize their binding mode. Finally, cinnamyl cinnamate was selected for further biological test, since it is the compound with the highest concentration in the EO (Table S1) and it is able of potentially binding the greatest number of kinases among all selected MTAs. It turns out to be a very interesting lead compound with anti-cancer activity, suitable for further development.

## 2. Results and Discussion

Nowadays, in silico methods represent innovative approaches in the drug discovery process, mostly thanks to high-performance computing innovation. In fact, excluding the clinical trials, chemoinformatics and bioinformatics play a crucial role in every step of the drug discovery design [29]. The Essential Oil University (EOU) [30] website is a chemoinformatics database, providing precise information about the world of EOs, that constitute a heterogeneous group of complex mixtures of organic substances. In the last few years, EOs derived from plants have become very important for phytomedicine [31,32]. Their widespread use has increased the attention of scientists in EOs field. Particularly, their anti-microbial, anti-oxidant, and anti-cancer activities have been investigated during the last years [33,34].

Cancer is the second largest single cause of death worldwide [35] and over the next twenty years is expected to rise by about 70%. The modern therapies have improved cancer patients’ life and chemoprevention with natural compounds is an emerging strategy to avoid and cure the tumor [3]. Different studies on plants and natural compounds have led to the discovery of many drugs, such as taxol, vincristine, vinblastine, and camptothecin [36,37]. Moreover, over five hundred scientific studies have been published on anti-cancer activity of EOs compounds [38,39,40,41]. Until now, the effects of EOs have been investigated on different types of cancer such as glioblastoma, melanoma, leukemia and cervix, colon, kidney, prostate, and uterus cancers. In light of these evidences, in this study we have considered EOs as new resources of multi-targeting compounds to screen towards different kinds of Protein Kinase targets, in order to better explain their mechanism of action.

### 2.1. Structure-Based Virtual Screening (SBVS)

In order to perform a SBVS, 3D structures were downloaded from the EOU website, as already reported in previous work [42]. The present study allowed us to select 13 compounds extracted from EOs, as potential agents with antiproliferative activity by binding multiple kinase targets (Table 1).

Among them, we focused our analysis on two natural compounds, cinnamyl cinnamate and α-terpinen-7-al, as those with higher percentages in the essential oil of *Liquidambar styraciflua L.* (fam. *Hamamelidaceae*) and *Cuminum cyminum L.* (fam. *Apiaceae*), respectively (Table S1). Cumin is a small, slender, erect glabrous annual herb with strong antioxidant activity and it is cultivated all over the world. Cumin has been reported to have antibacterial, antispasmodic, stomachic, carminative, antimicrobial, antifungal, larvicidal, antiseptic, antidiabetic, anticarcinogenic, cholesterol-lowering, anti-inflammatory, and antioxidant properties [43,44,45]. Cancer chemopreventive potentials of different doses of a cumin seed-mixed diet were evaluated against benzo(α)pyrene [B(α)P]-induced forestomach tumorigenesis and 3-methylcholanthrene (MCA)-induced uterine cervix tumorigenesis [46]. In addition, at a concentration of 0.1 μL/mL, cumin oil destructed Hela cells by 79%.

#### Cinnamyl Cinnamate and α-Terpinen-7-al

**Figure 1:**
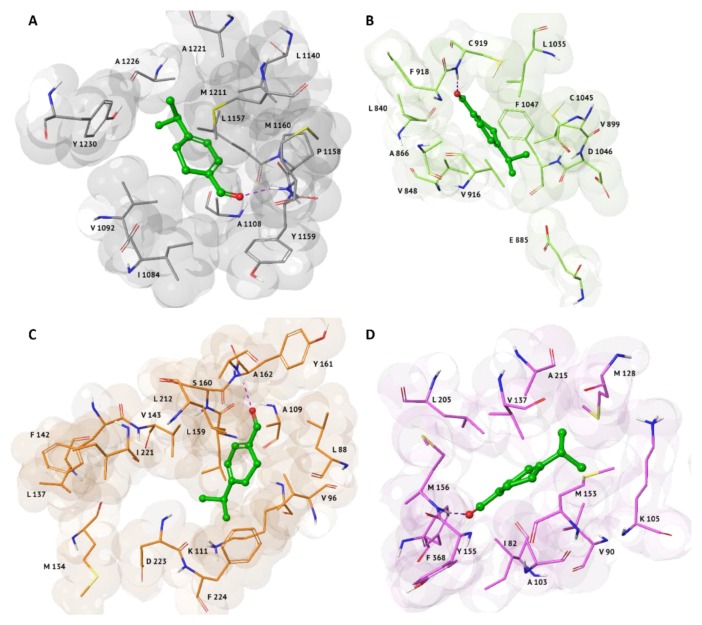
3D representations of the best docking pose of α-terpinen-7-al with the following anti-cancer targets: (**A**) c-Met (PDB: 2WGJ), represented as grey surface; (**B**) VEGFR2 (PDB: 3VHE), represented as green surface; (**C**) PDK1 (PDB: 3NAX), represented as orange surface; (**D**) ROCK1 (3TWJ), represented as pink surface. Ligands are shown in green carbon ball-and-sticks, while amino-acid residues involved in the molecular interactions are shown as carbon sticks with the correspondent color of protein’s surface. H-bonds is displayed as purple dashed lines.

SBVS results showed a good theoretical binding affinity of the α-terpinen-7-al compound towards four kinases, involved in cancer pathology: hepatocyte growth factor receptor (c-Met), vascular endothelial growth factor receptor 2 (VEGFR2), 3-phosphoinositide-dependent protein kinase 1 (PDK1), and Rho-associated protein kinase 1 (ROCK1) (Figure 1). In particular, for these four complexes, the G-score values range between −8.00 and −8.38 kcal/mol (Table 1). The main feature of the α-terpinen-7-al binding mode in all complexes is characterized by an H-bond between its carbonyl group and the amine group of backbone of the M1160, C919, A162 and M156 residues, associated with c-Met (Figure 1A), VEGFR2 (Figure 1B), PDK1 (Figure 1C) and ROCK1 (Figure 1D) pockets, respectively. Furthermore, the four complexes are well-stabilized thanks to numerous hydrophobic interactions with the following residues: L1157, A1226, A1221, and Y1230 for C-Met, F1047, V916 and L1035 for VEGFR2, A109, L159, F224, V96, and L88 for PDK1 and V137, M153, and M128 for ROCK1.

On the other hand, literature data reported an antioxidant and anti-cancer activity assessed as ABTS radical scavenging activity for the cinnamyl cinnamate [47]. Indeed, EOs contained into the leaves and the stems of *Liquidambar styraciflua L.* induce low cytotoxic activity on cervix cancer [48]. Lester A. Mitscher et al. illustrated the isolation of cinnamic acid, cinnamyl cinnamate and cinnamyl ricinoleate and their activity as antimutagenic agents [49].

**Figure 2:**
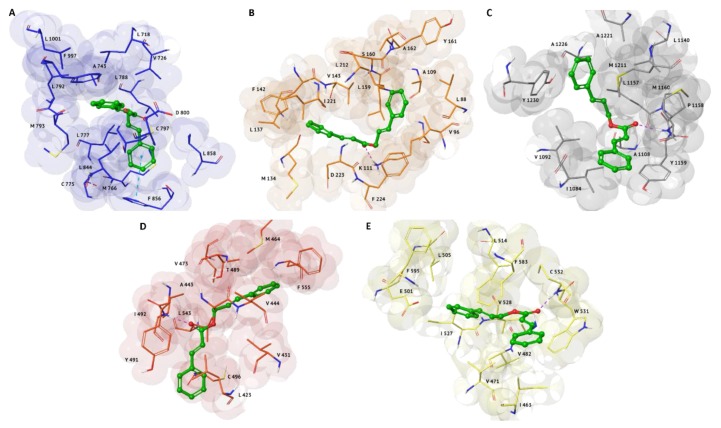
3D representations of the best docking pose of cinnamyl cinnamate with the following anti-cancer targets: (**A**) EGFR (PDB: 3POZ), represented as blue surface; (**B**) PDK1 (PDB: 3NAX), represented as orange surface; (**C**) c-Met (PDB: 2WGJ), represented as grey surface; (**D**) BMX (PDB: 3SXR), represented as red surface; (**E**) B-Raf (PDB: 2FB8), represented as yellow surface. Ligands are shown in green carbon ball-and-sticks, while amino-acid residues involved in the molecular interactions are shown as carbon sticks with the correspondent color of protein’s surface. H-bonds and π-π interactions are displayed as purple and cyan dashed lines, respectively.

Our theoretical results indicated this compound as a potential ligand *versus* five kinase structures: epidermal growth factor receptor (EGFR), PDK1, c-Met, cytoplasmic tyrosine-protein kinase (BMX) and B-Raf proto-oncogene serine/threonine-protein kinase (B-Raf) (Figure 2). As for α-terpinen-7-al, cinnamyl cinnamate showed a similar binding affinity for these targets, with G-score values ranging from −8.02 to −8.54 kcal/mol (Table 1). In particular, its binding mode with EGFR is characterized by the formation of a π-π interaction between the phenyl-allyl moiety and the side chain of P856 residue (Figure 2A). Regarding the other complexes, we observed the establishment of an H-bond between the carbonyl group of the ligand and the backbone amide group of M1160, I492, and C352, exposed in the binding pocket of c-Met, BMX and B-Raf, respectively (Figure 2C–E). Also PDK1 complex is characterized by the formation of an H-bond, that is engaged between the carbonyl group of cinnamyl cinnamate and the side chain of K111 residue (Figure 2B).

All the cinnamyl cinnamate-protein complexes, were stabilized by different hydrophobic interactions with F997, L788, L772, and L844 for EGFR; A109, V96, L159, and F224 for PDK1; Y1230, L1157, Y1159 and I1084 for c-Met; Y491, L423, V444, and F555 for BMX; W531, V482, F583, and I527 for B-Raf.

**Figure 3:**
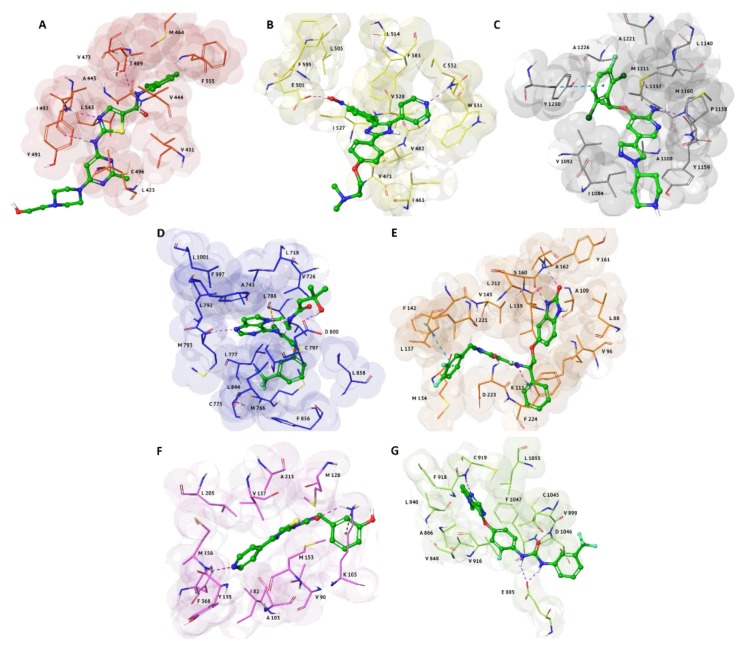
Re-docked best poses of the relative co-crystallized ligands in the Protein Data Bank (PDB) models: (**A**) BMX (PDB code: 3SXR), represented as red surface; (**B**) B-Raf (PDB code: 2FB8), represented as yellow surface; (**C**) c-Met (PDB code: 2WGJ), represented as grey surface; (**D**) EGFR (PDB code: 3POZ), represented as blue surface; (**E**) PDK1 (PDB code: 3NAX), represented as orange surface; (**F**) ROCK1 (PDB code: 3TWJ), represented as pink surface; (**G**) VEGFR2 (PDB code: 3VHE), represented as green surface. Ligands are shown in green carbon ball-and-sticks, while amino-acid residues involved in the molecular interactions are shown as carbon sticks with the correspondent color of protein’s surface. H-bonds, π-π and halogen interactions are displayed as purple, cyan and yellow-gold dashed lines, respectively.

The analysis of the best docking poses of MTAs selected in our SBVS pointed out comparable molecular interactions respect to each co-crystallized inhibitor (Table S2), also sharing the same amino-acid residues characteristic of each pocket (Figure 3). Only in c-Met we observed the involvement of a different residue for the formation of the H-bond that characterizes the binding mode of MTAs and the co-crystallized ligand, crizotinib. In fact, in X-ray model crizotinib engages an H-bond donor between the aniline group and the backbone carbonyl portion of P1158 (Figure 3C). On the contrary, both MTAs establish an H-bond acceptor involving the backbone amide portion of M1160 (Figure 1A and Figure 2C).

In particular, a very similar binding mode, characterized by an H-bond with I492, is observed in cinnamyl cinnamate and dasatinib complexes with BMX (Figure 2D and Figure 3A).

Likewise, the involvement of two H-bonds with A162 and K111 are similar for α-terpinen-7-al, cinnamyl cinnamate and the co-crystallized inhibitor in PDK1 complexes (Figure 1C, Figure 2B and Figure 33E), respectively.

Regarding VEGFR2 target, the co-crystallized pyrrolopyrimidine derivative and α-terpinen-7-al shared one H-bond with C919 (Figure 3G and Figure 1B), while in ROCK1 complexes, the interaction with M156 resulted pivotal (Figure 1D and Figure 3F).

Finally, the cinnamyl cinnamate complexed to B-Raf (Figure 2E) is well stabilized into the binding pocket through the same H-bond acceptor with the backbone amide portion of C532 residue (Figure 3B).

### 2.2. Induced Fit Docking

Finally, the two compounds with higher percentages in the EOs were further studied by using the Schrödinger’s Induced Fit Docking (IFD) protocol, that predicts ligand binding modes, remarkably considering the conformational changes induced by the bound ligand in the receptor pocket [50]. IFD results confirmed the good affinity of both compounds cinnamyl cinnamate and α-terpinen-7-al towards the kinase targets selected by docking simulations, as reported in Table 2. As comparison, in Table S3 we reported both Glide and IFD scores of each co-crystallized ligand in X-ray kinase models. By analyzing the best IFD poses for all α-terpinen-7-al complexes, we observed the same binding mode indicated by Glide SP protocol and an improved ligand recognition due to additional hydrophobic interactions (Figure S1). Conversely, regarding cinnamyl cinnamate, we examined a different binding mode in EGFR and c-Met pockets (Figure S2A,E), while observing the same docking interactions in PDK1, BMX and B-Raf complexes (Figure S2B–D). In particular, the best IFD pose of cinnamyl cinnamate towards EGFR (Figure S2A) is characterized by an H-bond between its carbonyl group and the side chain of K745. On the other hand, in the c-Met complex (Figure S2E), the cinnamyl cinnamate is able to engage an H-bond between its carbonyl group and the amide function of D1222 backbone, and a stacking interaction with Y1230.

In order to perform biological tests, we decided to purchase the cinnamyl cinnamate, as the compound with the higher concentration in the EOs (Table S1) and with a good binding affinity *versus* 5 different kinases.

### 2.3. Cell Viability Assay

To investigate the biological effects of the cinnamyl cinnamate, we performed an antiproliferative cell titer assay on a panel of human cancer cell lines from several tumors including MCF7 (breast adenocarcinoma), A549 (lung cancer), HL60 (leukemia), and Hela (cervical cancer). We assessed the most significant effect on cell viability of MCF7, A549, HL60 cells forty-eight hours after cinnamyl cinnamate administration.

Interestingly, on MCF7 the effect was time-dependent, while in the other cell lines the reduction of cell proliferation decreased seventy-two hours after treatment. The effect on HeLa cells could only be appreciated after seventy-two hours following the compound use.

**Figure 4:**
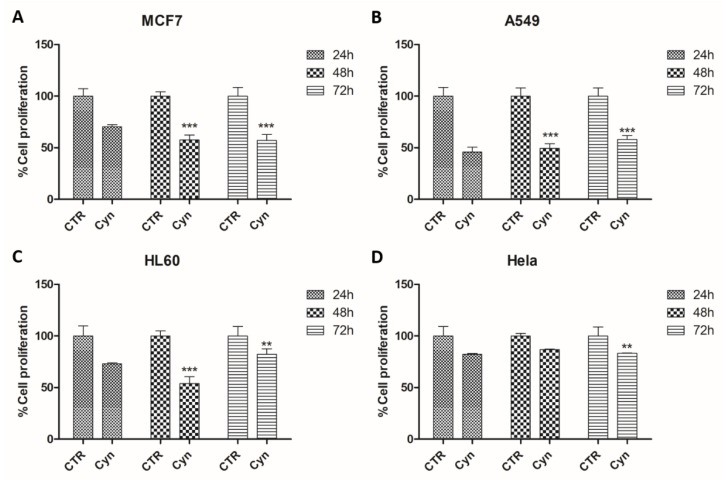
Effects of cinnamyl cinnamate treatment on cell proliferation of (**A**) MCF7, (**B**) A549, (**C**) HL60, and (**D**) Hela malignant cells. 500 cells were plated, in triplicate, in 384-well plates and treated with cinnamyl cinnamate at concentration of 10 μM for 24, 48 and 72 h; cells treated with DMSO were used as control. Cell proliferation was measured performing a cell titer assay and expressed as a percentage of control (** p < 0.005; *** p < 0.0005). Each column represents the mean ± SD of three different wells.

Therefore, our data propose cinnamyl cinnamate as an interesting lead compound with antiproliferative effect suitable for further development (Figure 4).

## 3. Conclusions

Virtual screening experiments represent a fast and cheap approach able to identify novel multi-targets *hit* compounds with a polypharmacological profile. In this study, we applied computational methods in order to discover new potential anti-cancer scaffolds obtained from natural products, in particular from the EOs, that could be considered a new and sustainable resource of kinase inhibitors. Indeed, a recent review [51] has just collected the anti-cancer activities of different bioactive components of many EOs, such as terpenes and phenylpropanoids (carvacrol, cinnamaldehyde, eugenol, α-terpineol, and thymol), which can exert multiple effects on specific cancer mechanisms. Thus, these compounds could have a huge potential to improve patients’ quality of life and to increase their chances of survival. Some of the compounds mentioned in this review were just identified through our theoretical study, thus representing a further example of the proven predictive capacity of the Computer-Aided Drug Design (CADD) methodologies [52]. Cell viability assay confirms the anti-cancer activity of the cinnamyl cinnamate that can be considered as an interesting lead compound suitable for further development. To date, the EOs bioactive compounds are available online on an accessible web-based platform, the Mu.Ta.Lig. Virtual Chemotheca [53,54,55].

## 4. Materials and Methods

### 4.1. Ligands Database Preparation

The chemical structures of 2690 compounds were downloaded from the EOU website [30]. Lig-prep module implemented in Schrödinger Suite ver. 11 was used to calculate the protonated and tautomeric form of all compounds at pH 7.4 and to optimize all ligands [56]. The ZINC15 algorithm was used in order to identify Pan Assays Interference (PAINS) compounds [57,58], that could influence the identification of new bioactive compounds with false activity signals. We obtained a library of 2304 compounds and submitted to virtual screening.

### 4.2. Targets Preparation

The 3D coordinates of 23 Protein Kinases targets were selected from the Protein Data Bank (PDB) platform [59]. The criteria for the X-ray targets selection were: (1) PDB deposition data; (2) origin from *Homo Sapiens*; (3) presence of one co-crystallized ligand; (4) crystallographic resolution <3.50 Å; (5) ability to reproduce the experimental complex using the re-docking procedure evaluating the Root Mean Square Deviation (RMSD) values (Table S4). For each selected X-ray model, the Protein Preparation Wizard tool was used in order to add hydrogens, to assign partial charges, to build missing atoms, side chains and loops [60]. The resulting structures were submitted to energy optimization by using a specific workflow already reported in a previous study [42].

### 4.3. Glide Docking

Docking studies were carried out by using Glide Standard Precision (SP) protocol, implemented in Glide ver. 7.2 [61], with default parameters, generating ten poses for each ligand. Rigid receptor grid defined by a 10 × 10 × 10 Å inner box was generated. In particular, each co-crystallized ligand of PDB models was used to center the docking grid box and to check the prediction of the binding affinity and geometry. Thus, in order to assess the docking reliability, re-docking simulations were carried out, observing a good capability of the docking software to reproduce the experimental pose of the co-crystallized inhibitor. RMSD values were adopted to investigate differences between the inhibitor crystallographic geometry and the best pose generated by docking simulations (Table S4).

Finally, EO library was screened towards all 23 protein kinases and the most promising compounds, with the best G-score value, were selected using a *cut-off* of −8.00 kcal/mol, as reported in a previous work [17].

### 4.4. Induced Fit Docking Protocol

The best docking poses of the most abundant compounds found in nature (Table S1), cinnamyl cinnamate and α-terpinen-7-al, were submitted to Induced Fit Docking protocol using the Standard Protocol and generating 20 poses for ligand, in order to better rationalize their binding interactions.

### 4.5. Cell Viability Assay

A549 (lung cancer), HL60 (leukaemia), and Hela (cervical cancer) human cell lines were cultured in RPMI medium (Sigma Aldrich, St. Louis, MO, USA) supplemented with 10% fetal bovine serum (FBS) (Sigma Aldrich, St. Louis, MO, USA), 1% Penicillin/Streptomycin (Sigma Aldrich, St. Louis, MO, USA).

MCF7 (breast cancer) and M14 (melanoma) human cell lines, were cultured in DMEM medium (Sigma Aldrich, St. Louis, MO, USA) supplemented with 10% fetal bovine serum (FBS) (Sigma Aldrich, St. Louis, MO, USA), 1% Penicillin/Streptomycin (Sigma Aldrich, St. Louis, MO, USA). Cells were grown in a 5% CO_2_ incubator at 37 °C.

Five hundred cells were seeded in a 384-well plate and treated with cinnamyl cinnamate (dissolved in DMSO) at concentration of 10 μM. The treatment was dispensed by Echo 550 Liquid Handler (10 nL). Every twenty-four hours, a Cell Titer Glo detection reagent (Promega Corp, Madison, WI, USA), were added, and plate gently mixed for 10 min in the dark. The absorbance was measured by EnVision Multilabel 2103.

## Figures and Tables

**Table 1:** Name, CAS (Chemical Abstracts Service) number and 2D structure of essential oils (EOs) components selected after Structure-Based Virtual Screening (SBVS) are shown together with the Protein Kinases targets and their related best theoretical affinities (G-score in kcal/mol).

Name CAS Number	Structure	Target	G-Score
Psoralen CAS 66-97-7	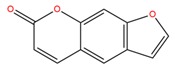	VEGFR2	−8.09
		c-Met	−8.46
1-H-indol-2-ol CAS 16990-73-1	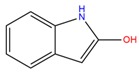	VEGFR2	−8.00
		PDK1	−8.64
α-terpinen-7-al CAS 1197-15-5	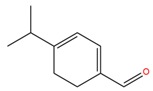	VEGFR2	−8.38
		PDK1	−8.15
		ROCK1	−8.00
		c-Met	−8.16
Hinokitiol CAS 499-44-5	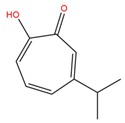	VEGFR2	−8.42
		c-Met	−8.20
β-Vetivone CAS 18444-79-6	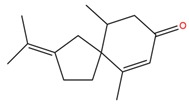	ROCK1	−8.05
		c-Met	−8.26
Precocene II CAS 644-06-4	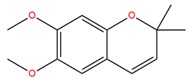	VEGFR2	−8.02
		MEK2	−8.08
1-H-benzochromene CAS 5153-92-4	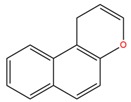	VEGFR2	−8.18
		PI3K-γ	−8.95
		c-Met	−8.88
Piperonylacetone CAS 55418-52-5	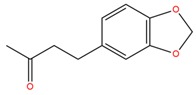	VEGFR2	−8.36
		ROCK1	−8.33
		c-Met	−9.10
Thymohydroquinone CAS 2217-60-9	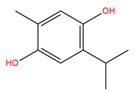	SGK1	−8.57
		VEGFR2	−8.70
		PDK1	−8.66
		CDK2	−8.00
		GSK3β	−8.13
		ERK2	−8.18
3-Phenil Benzaldeide CAS 1204-60-0	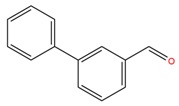	VEGFR2	−9.47
		MEK2	−8.41
		ROCK1	−8.35
		B-Raf	−8.10
		c-Met	−8.07
Cinnamyl cinnamate CAS 122-69-0	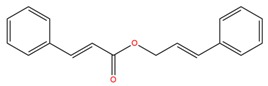	EGFR	−8.12
		PDK1	−8.19
		BMX	−8.06
		B-Raf	−8.02
		c-Met	−8.54
Isoquinoline CAS 119-65-3	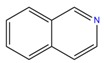	VEGFR2	−8.23
		PKA	−8.00
		ROCK1	−8.04
		c-Met	−8.12
Atronorin CAS 479-20-9	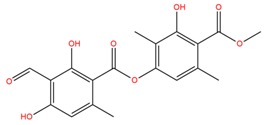	SGK1	−8.02
		EGFR	−9.69
		VEGFR2	−10.97
		ABK	−8.17
		B-Raf	−8.01
		ERK1	−9.06
		c-Met	−8.46

**Table 2:** G-score and Induced Fit Docking (IFD) scores, expressed in kcal/mol, of α-terpinen-7-al and cinnamyl cinnamate complexed with kinase proteins selected by SBVS.

Name CAS Number	Target	G-Score	IFD Score
α-terpinen-7-al CAS 1197-15-5	VEGFR2	−8.82	−665.28
	PDK1	−8.52	−603.38
	ROCK1	−8.18	−870.20
	c-Met	−8.62	−633.60
Cinnamyl cinnamate CAS 122-69-0	EGFR	−8.31	−654.13
	PDK1	−9.46	−605.92
	BMX	−8.72	−575.64
	B-Raf	−8.64	−563.84
	c-Met	−9.50	−635.95

## Supplementary Materials

The following are available online. Table S1. Percentage of compounds contained in essential oils (EOs); Table S2. For each analysed target: the PDB accession code, the name of the co-crystallized ligand and the relative binding affinity G-score value (in kcal/mol) after re-docking simulations; Table S3. G-score and IFD scores, expressed in kcal/mol, of PDB co-crystallized ligands complexed with their related kinase proteins; Figure S1. 3D representations of best IFD pose of α-terpinen-7-al with the following anti-cancer targets: (A) VEGFR2 (PDB: 3VHE), represented as green surface; (B) PDK1 (PDB: 3NAX), represented as orange surface; (C) ROCK1 (3TWJ), represented as pink surface; (D) c-Met (PDB: 2WGJ), represented as grey surface. Ligands are shown in green carbon ball-and-sticks, while amino acid residues involved in the molecular interactions are shown as carbon sticks with the correspondent color of protein’s surface. H-bonds and π-π interactions are displayed as purple and cyan dashed lines, respectively; Figure S2. 3D representations of best IFD pose of cinnamyl cinnamate with the following anti-cancer targets: (A) EGFR (PDB: 3POZ), represented as blue surface; (B) PDK1 (PDB: 3NAX), represented as orange surface; (C) BMX (PDB: 3SXR), represented as red surface; (D) B-Raf (PDB: 2FB8), represented as yellow surface; (E) c-Met (PDB: 2WGJ), represented as grey surface. Ligands are shown in green carbon ball-and-sticks, while amino acid residues involved in the molecular interactions are shown as carbon sticks with the correspondent color of protein’s surface. H-bonds and π-π interactions are displayed as purple and cyan dashed lines, respectively; Table S4. Target and abbreviations of the 23 Protein Kinases targets, selected for the SBVS study, with the relative PDB accession code and X-ray resolution expressed in Å. For each target, the RMSD value, expressed in Å, was calculated after re-docking simulation between the experimental pose of the co-crystallized ligand and the best pose generated by the docking protocol. The binding energy of the re-docked pose was reported as G-score value, expressed in kcal/mol.
