# Supplementary material for: Multi-Targeting Bioactive Compounds Extracted from Essential Oils as Kinase Inhibitors
Source: Molecules. 2020 May 6;25(9):2174. doi: 10.3390/molecules25092174 (PMC7249159; doi:10.3390/molecules25092174)
Supplement: Supplementary file 1 [file molecules-25-02174-s001.pdf]

Supplementary Material

Multi-Targeting bioactive compounds extracted from Essential Oils as kinase inhibitors

Annalisa Maruca [1,2], Delia Lanzillotta [3], Roberta Rocca [2,3], Antonio Lupia [2], Giosuè Costa [1,2], Raffaella Catalano [1,2], Federica Moraca [2,4], Eugenio Gaudio [5], Francesco Ortuso [1,2], Anna Artese [1,2], Francesco Trapasso [3], Stefano Alcaro [1,2,*]

[1] Dipartimento di Scienze della Salute, Università "Magna Græcia" di Catanzaro, Campus Universitario "S. Venuta", Viale Europa, Loc. Germaneto, 88100 Catanzaro, Italy.

[2] Net4Science srl, Università "Magna Græcia" di Catanzaro, Campus Salvatore Venuta, Viale Europa, 88100, Catanzaro, Italy.

[3] Department of Experimental and Clinical Medicine, Università "Magna Græcia" di Catanzaro, Campus Universitario "S. Venuta", Viale Europa, Loc. Germaneto, 88100 Catanzaro, Italy.

[4] University "Federico II" of Naples, Department of Pharmacy, Via D. Montesano 49, Naples, 80131, Italy.

[5] Lymphoma and Genomics Research Program, The Institute of Oncology Research, Bellinzona, Switzerland.

*Correspondence: Stefano Alcaro alcaro@unicz.it +39 0961 3694198 / +39 0961 391490

Received: date; Accepted: date; Published: date

Table S1. Percentage of compounds contained in essential oils (EOs).

Oil Name	Compounds	Botanic name	Percentage (%)
**Styrax**	**Cinnamyl cinnamate**	*Liquidambar styraciflua* L., fam. Hamamelidaceae	38.00
**Cumin**	**α-terpinen-7-al**	*Cuminum cyminum* L., fam. Apiaceae (Umbelliferae)	17.77
**Ageratum conyzoides**	**Precocene II**	*Ageratum conyzoides* L., fam. Asteraceae	17.65
**Vetiver**	**β-vetivone**	*Vetiveria zizanioides* Stapf, fam. Poaceae (Gramineae)	5.00
**Parsley leaf**	**Psoralen**	*Petroselinum crispum*, fam. Apiaceae	3.20
**Rue 1**	**Piperonylacetone**	*Ruta graveolens* L., fam. Rutaceae	3.10
**Oregano**	**Thymohydroquinone**	*Coridothymus capitatus* Rchb., fam. Lamiaceae	1.40
**Jasmine sambac**	**1H-indol-2ol**	*Jasminum sambac* (L.) Aiton, fam. Oleaceae	0.50
**Cassie absolute**	**Isoquinoline**	*Acacia farnesiana* (L.) Willd., fam. Mimosaceae	0.50
**Oakmoss**	**Atronorin**	*Evernia prunastri* (L.) Ach., fam. Usneaceae	0.30
**Vassoura**	**1H-benzochromene**	*Baccharis dracunculifolia* DC., fam. Asteraceae	0.20
**Star anise**	**Hinokitiol**	*Illicium verum* Hooker fil., fam. Magnoliaceae	0.19
**Ginger**	**3-Phenylbenzaldehyde**	*Zingiber officinale* Roscoe, fam. Zingiberaceae	0.01

Table S2. For each analysed target: the PDB accession code, the name of the co-crystallized ligand and the relative binding affinity G-score value (in kcal/mol) after re-docking simulations.

Target	PDB code	Co-crystallized ligand	G-score
**c-Met**	2WGJ	Crizotinib	-12.03
**VEGFR2**	3VHE	Pyrrolopyrimidine derivative	-13.90
**EGFR**	3POZ	Tak-285	-13.89
**B-Raf**	2FB8	SB-590885	-10.95
**PDK1**	3NAX	1-(3,4-difluorobenzyl)-2-oxo-N-{(1R)-2-[(2- oxo-2,3-dihydro-1H-benzimidazol-5-yl)oxy]- 1-phenylethyl}-1,2-dihydropyridine-3-carboxamide	-18.82
**BMX**	3SXR	Dasatinib	-11.29
**ROCK1**	3TWJ	RKI1447	-8.93

Table S3. G-score and IFD scores, expressed in kcal/mol, of PDB co-crystallized ligands complexed with their related kinase proteins.

Target	G-score	IFD Score
**VEGFR2**	-14.20	-877.00
**ROCK1**	-9.23	-870.20
**EGFR**	-15.87	-669.32
**PDK1**	-19.61	-621.81
**BMX**	-12.68	-587.91
**B-Raf wt**	-10.15	-574.98
**c-Met**	-14.45	-643.86

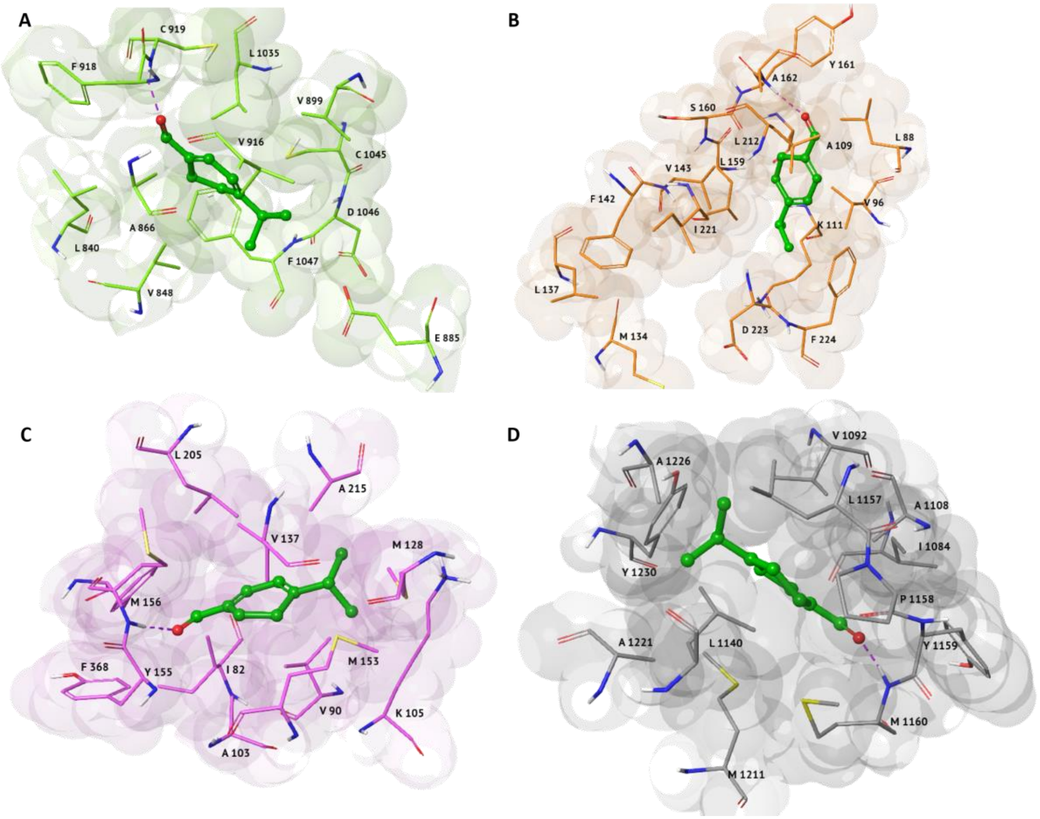

Figure S1. 3D representations of the best IFD pose of α-terpinen-7-al with the following anti-cancer targets: (**A**) VEGFR2 (PDB: 3VHE), represented as green surface; (**B**) PDK1 (PDB: 3NAX), represented as orange surface; (**C**) ROCK1 (3TWJ), represented as pink surface; (**D**) c-Met (PDB: 2WGJ), represented as grey surface. Ligands are shown in green carbon ball-and-sticks, while amino acid residues involved in the molecular interactions are shown as carbon sticks with the correspondent color of protein's surface. H-bonds and π-π interactions are displayed as purple and cyan dashed lines, respectively.

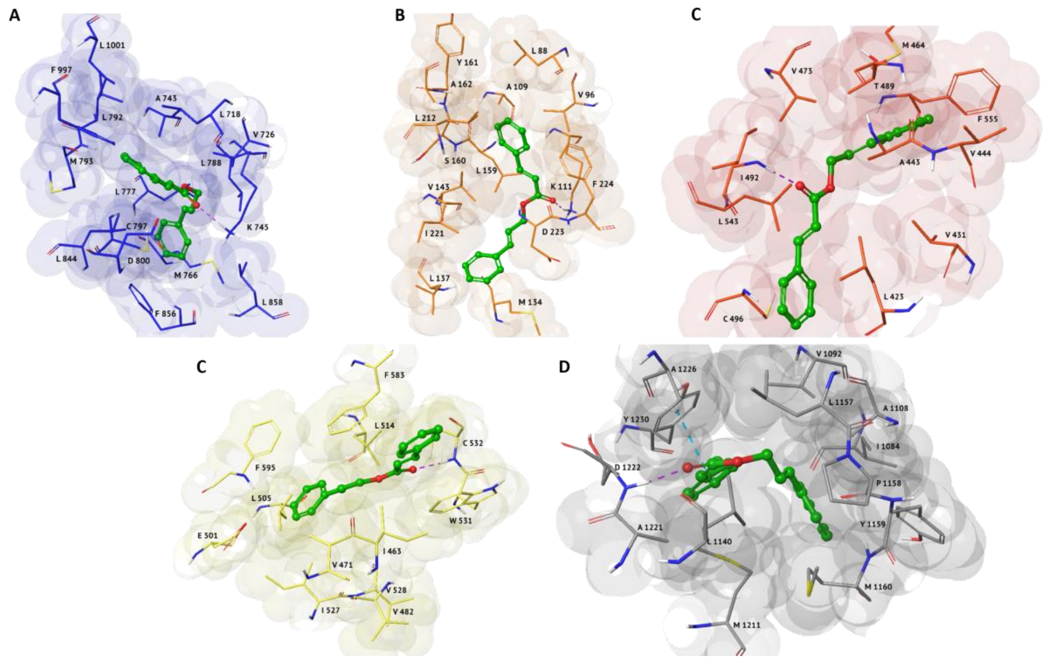

Figure S2. 3D representations of the best IFD pose of cinnamyl cinnamate with the following anti-cancer targets: (**A**) EGFR (PDB: 3POZ), represented as blue surface; (**B**) PDK1 (PDB: 3NAX), represented as orange surface; (**C**)

BMX (PDB: 3SXR), represented as red surface; (**D**) B-Raf (PDB: 2FB8), represented as yellow surface; (**E**) c-Met (PDB: 2WGJ), represented as grey surface. Ligands are shown in green carbon ball-and-sticks, while amino acid residues involved in the molecular interactions are shown as carbon sticks with the correspondent color of protein's surface. H-bonds and π-π interactions are displayed as purple and cyan dashed lines, respectively.

Table S4. Target and abbreviations of the 23 Protein Kinases targets, selected for the SBVS study, with the relative PDB accession code and X-ray resolution expressed in Å. For each target, the RMSD value, expressed in Å, was calculated after re-docking simulation between the experimental pose of the co-crystallized ligand and the best pose generated by the docking protocol. The binding energy of the re-docked pose was reported as G-score value, expressed in kcal /mol.

Target	Abbreviation	PDB models	Resolution	RMSD	G-score
**Serine/threonine-protein kinases**	Akt	4GV1	1.49	1.06	-11.63
**Aurora A kinase**	AAK	2X81	2.91	0.47	-9.38
**Aurora B kinase**	ABK	2VRX	1.86	0.74	-10.18
**Cytoplasmic tyrosine-protein kinase**	BMX	3SXR	2.40	0.89	-11.29
**B-Raf proto-oncogene serine/threonine-protein kinase**	B-Raf	2FB8	2.90	0.68	-10.95
**B-Raf proto-oncogene serine/threonine-protein kinase V600E**	B-Raf V600E	3OG7	2.45	1.62	-12.24
**Cyclin-dependent kinase 2**	CDK2	4KD1	1.70	1.39	-11.61
**Hepatocyte growth factor receptor**	c-Met	2WGJ	1.20	0.85	-12.03
**Epidermal growth factor receptor**	EGFR	3POZ	1.50	0.89	-13.89
**Epidermal growth factor receptor V948R**	EGFR V948R	4I22	1.71	1.01	-8.71
**Extracellular signal regulated Kinases 1**	ERK1	2ZOQ	2.39	0.37	-9.26
**Extracellular signal regulated Kinases 2**	ERK2	4ZZN	1.45	0.58	-12.41
**Glycogen synthase kinase-3β**	GSK3β	4ACC	2.21	1.20	-9.15
**Kinesin-like protein KIF11**	Kinesis Eg5	2X7C	1.90	0.12	-10.31
**Mitogen-activated protein kinase 1**	MEK1	4ARK	2.60	0.71	-8.64
**Mitogen-activated protein kinase 2**	MEK2	1S9I	3.20	0.38	-8.61

**3-Phosphoinositide-dependent protein kinase 1**	PDK1	3NAX	1.75	0.34	-18.82
**Phosphoinositide-3-kinase α**	PI3Kα	5UBR	2.40	0.92	-10.48
**Phosphoinositide-3-kinase γ**	PI3Kγ	3DBS	2.80	0.78	-9.34
**cAMP-dependent protein kinase**	PKA C-α	3POO	1.60	0.23	-12.35
**Rho-associated protein kinase 1**	ROCK1	3TWJ	2.90	0.55	-8.98
**Serum and glucocorticoid-regulated kinase 1**	SGK1	3HDM	2.60	0.68	-9.77
**Vascular endothelial growth factor receptor 2**	VEGFR2	3VHE	1.55	0.34	-13.90
